# *In Vitro* Effects of Pirfenidone on Cardiac Fibroblasts: Proliferation, Myofibroblast Differentiation, Migration and Cytokine Secretion

**DOI:** 10.1371/journal.pone.0028134

**Published:** 2011-11-23

**Authors:** Qiang Shi, Xiaoyan Liu, Yuanyuan Bai, Chuanjue Cui, Jun Li, Yishi Li, Shengshou Hu, Yingjie Wei

**Affiliations:** State Key Laboratory of Cardiovascular Disease, National Center for Cardiovascular Disease, Fuwai Hospital, Chinese Academy of Medical Sciences, Peking Union Medical College, Beijing, People Republic China; Harvard Medical School, United States of America

## Abstract

Cardiac fibroblasts (CFs) are the primary cell type responsible for cardiac fibrosis during pathological myocardial remodeling. Several studies have illustrated that pirfenidone (5-methyl-1-phenyl-2-[1H]-pyridone) attenuates cardiac fibrosis in different animal models. However, the effects of pirfenidone on cardiac fibroblast behavior have not been examined. In this study, we investigated whether pirfenidone directly modulates cardiac fibroblast behavior that is important in myocardial remodeling such as proliferation, myofibroblast differentiation, migration and cytokine secretion. Fibroblasts were isolated from neonatal rat hearts and bioassays were performed to determine the effects of pirfenidone on fibroblast function. We demonstrated that treatment of CFs with pirfenidone resulted in decreased proliferation, and attenuated fibroblast α-smooth muscle actin expression and collagen contractility. Boyden chamber assay illustrated that pirfenidone inhibited fibroblast migration ability, probably by decreasing the ratio of matrix metalloproteinase-9 to tissue inhibitor of metalloproteinase-1. Furthermore, pirfenidone attenuated the synthesis and secretion of transforming growth factor-β1 but elevated that of interleukin-10. These direct and pleiotropic effects of pirfenidone on cardiac fibroblasts point to its potential use in the treatment of adverse myocardial remodeling.

## Introduction

Structural remodeling of the left ventricle, which is initiated by pathological events such as hypertension or myocardial infarction, can ultimately lead to heart failure (HF). Adverse myocardial remodeling is characterized by fibrosis, myocyte death, hypertrophy of surviving myocytes, and proliferation of cardiac fibroblasts (CFs) [1]. CFs are the most abundant cell type present in the myocardium and play a key role in maintaining its structural integrity through controlled proliferation and extracellular matrix (ECM) turnover, CFs are therefore perceived as the primary cell type responsible for cardiac fibrosis during adverse myocardial remodeling [2]–[5]. In response to pathological stimuli, CFs undergo a phenotypic transformation to become cardiac myofibroblasts that express contractile proteins. Cardiac myofibroblasts are highly proliferative and migrative, and remodel the cardiac interstitium by increasing secretion of matrix-degrading metalloproteinases (MMPs). To stimulate the remodeling process further, they secrete increased amounts of growth factors and cytokines, such as transforming growth factor (TGF)-β1, interleukin (IL)-6 and tumor necrosis factor (TNF)-α [6]–[8]. Although these changes serve initially as an important reparative wound healing response, in the longer term, they become maladaptive and lead to abnormal myocardial stiffness and ultimately, ventricular dysfunction.

Pirfenidone (5-methyl-1-phenyl-2-[1H]-pyridone) is a small molecule that inhibits progression of fibrosis *in vivo* in a variety of animal models of lung [9]–[11], kidney [12], [13], hepatic [14] and cardiac fibrosis [13], [15]–[17]. *In vitro* studies have shown that pirfenidone inhibits proliferation and/or activation of a wide range of cell types including human lung fibroblasts [18], human myometrial and leiomyoma cells [19], human Tenon's fibroblasts [20], human T cells [21], rat hepatic stellate cells [22], and rat renal fibroblasts [23]. In addition, pirfenidone modulates a variety of cytokines, and it has been shown that it decreases levels of intercellular adhesion molecule-1 in cultured human synovial fibroblasts [24], inhibits heat shock protein 47 expression in human lung fibroblasts [25], downregulates TGF-β in human Tenon's fibroblasts [20], and suppresses translation of TNF-α in a murine macrophage-like cell line [26].

As mentioned above, it has been shown that pirfenidone attenuates cardiac fibrosis in several animal models, including a rat model of myocardial infarction [15], canine model of pacing-induced chronic heart failure [16], and a deoxycorticosterone acetate–salt hypertensive rat model [17]. Although results from these studies suggest that CFs represent the major targets of pirfenidone, however, to the best of our knowledge, no information is available regarding the effects of pirfenidone on cardiac fibroblast behavior. The aim of the present study was therefore to investigate the specific effects of pirfenidone on the cellular function of cultured CFs.

Here, we showed that pirfenidone effectively inhibited the proliferation, myofibroblast differentiation, collagen contraction, and migration of cardiac fibroblasts. We also found that pirfenidone reduced the ratio of MMP-9 to tissue inhibitor of metalloproteinase (TIMP)-1 in CFs. In addition, it decreased both mRNA expression and protein secretion of profibrotic cytokine, TGF-β1, but augmented that of anti-inflammatory cytokine, IL-10.

## Methods

### Ethics Statement

All procedures in the present study were conducted in accordance with the NIH Guide for the Care and Use of Laboratory Animals and approved by the Animal Care Committee of Cardiovascular Institute and Fuwai Hospital (Permit Number: 308).

### Reagents and chemicals

Dulbecco's modified Eagle's medium (DMEM), fetal calf serum (FCS), Trizol reagent, Novex 10% zymogram gels containing gelatin, renaturing buffer, developing buffer, and Colloidal Blue Staining Kit were purchased from Invitrogen (Carlsbad, CA, USA). AMV Reverse Transcriptase Kit and CellTiter 96® AQ_ueous_ One Solution Cell Proliferation Assay Kit were obtained from Promega (Madison, WI, USA). Power SYBR Green PCR Master Mix was from Applied Biosystems (Foster City, CA, USA). Rabbit anti-Ki67 monoclonal antibody was form Abcam (Cambridge, MA, USA). Alexa Fluor 488-conjugated anti-rabbit secondary antibody was from Molecular Probes (Eugene, OR, USA). The Fluorescein FragEL™ DNA Fragmentation Detection Kit was from Calbiochem (San Diego, CA, USA). ELISA detection kits for TIMP-1 and TGF-β1 were from R&D Systems (Minneapolis, MN, USA), and kit for IL-10 was from Ray Biotech (Norcross, GA, USA). Mouse anti-α-smooth muscle actin (α-SMA) monoclonal antibody and angiotensin II (Ang II) were from Sigma (St. Louis, MO, USA), monoclonal antibody against β-tubulin and horseradish peroxidise-conjugated anti-mouse secondary antibody were from Santa Cruz Biotechnology (Santa Cruz, CA, USA), rhodamine conjugated anti-mouse secondary antibody was from Proteintech Group (Chicago, IL, USA). Cell Contraction Assay Kit was from Cell Biolabs (San Diego, CA, USA). The Lactate Dehydrogenase (LDH) Cytotoxicity Detection Kit was from Jiancheng Bio-engineering Institute (Nanjing, China). Pirfenidone was from Yingxuan Chempharm (CAS No: 53179-13-8, Shanghai, China).

### Cell culture

CFs were obtained from the ventricles of neonatal Sprague–Dawley rats (1–3 days old) by the trypsin digestion method and characterized as previously described [27]. All experiments were performed in cells of the second and third passage after starvation in serum-free DMEM for 24 h.

### Cell proliferation assay

CF proliferation was assessed using the CellTiter 96® AQ_ueous_ One Solution Cell Proliferation Assay (MTS) Kit. Cells in exponential growth were harvested and plated in 96-well plates at a density of 5000 cells/well in 200 µl DMEM, incubated overnight, then starved by serum deprivation for 24 h, and treated with various concentrations (final concentrations: 0, 0.1, 0.5, 1.0, and 1.5 mg/ml; 0 mg/ml was designated as the control group) of pirfenidone in 10% FCS DMEM for 12, 24, 48, and 72 h. Twenty microliters of CellTiter 96® AQ_ueous_ One Solution reagent MTS, 3-(4,5-dimethylthiazol-2-yl)-5-(3-carboxymethoxyphenyl)-2-(4-sulfophenyl)-2H-tetrazolium, was added to each well, and cells were incubated for 1.5 h. Finally, the absorbance of the samples was measured at 490 nm using a model 680 microplate reader (Bio-Rad, Hercules, CA, USA). For cell number count, cell suspensions were seeded onto 6-well plates, treated with different concentrations of pirfenidone for 48 h. Then cells were resuspended and counted under the microscope.

### Collagen contraction assay

Collagen gel contraction mediated by CFs was evaluated using a Cell Contraction Assay Kit. Briefly, cells were harvested and resuspended in DMEM at 4×10^6^ cells/ml, and the collagen lattice was prepared by mixing two parts of cell suspension and eight parts of cold collagen gel solution. Subsequently, 500 µl of the cell–collagen mixture was cast into each well of a 24-well plate and allowed to polymerize at 37°C for 1 h. After collagen polymerization, cultures were incubated in DMEM for 2 days, during which stress developed. Pirfenidone was added to the culture medium at different concentrations (0, 1.0 and 1.5 mg/ml) and incubated for 24, 48 and 72 h. To initiate collagen contraction, polymerized gels were gently released from the walls of the wells. To determine the degree of collagen gel contraction, pictures of the gels were taken in flatbed scanner, and the area of each gel was analyzed with Quantity One software (Bio-Rad). Data were expressed as a percentage of the uncontracted gel size.

### Migration assay

CF migration assays were performed using a modified Boyden chamber technique with Matrigel basement membrane matrix-coated membranes (8 µm pore size, BD Biosciences, Bedford, MA, USA) as described previously [28]. Serum-starved CFs (10^5^) were loaded into the upper chamber of the migration apparatus. DMEM containing 0.25% FCS was introduced into the lower chamber as chemotactic stimulus. Pirfenidone at different concentrations (0, 0.5, 1.0 and 1.5 mg/ml) were added to the upper and lower chambers of the experimental wells. After incubation for 24 h at 37°C in a tissue culture incubator, inserts were collected and rinsed several times. Adherent non-migratory cells on the upper side of the membranes were rubbed off with a moist cotton swab. Migrated cells on the underside of the membrane were visualized by crystal violet staining and photographed using a light microscope (Olympus BX61, Tokyo, Japan). Migration was quantified by counting the invaded cells in 10 random high power fields (×400) for each membrane.

### RNA isolation and real-time PCR analysis

After being treated with 0, 0.5, 1.0 or 1.5 mg/ml pirfenidone for 48 h, total RNA was extracted from the CFs using Trizol reagent according to the manufacturer's instructions and quantified using UV spectrophotometry. cDNA was generated from 1 µg total RNA using an AMV Reverse Transcriptase Kit. Real-time PCR was performed in an Applied Biosystems 7300 Fast Real-Time PCR System (Foster City, CA, USA) with SYBR Green PCR Master Mix. Glyceraldehyde-3-phosphate dehydrogenase (GAPDH) mRNA amplified from the same samples served as an internal control. The relative expression of each targeted gene was normalized by subtracting the corresponding GAPDH threshold cycle (Ct) values using the ΔΔCt comparative method. The sequences of all primers used in this work are as follows: α-SMA: 5′-AGCCAGTCGCCATCAGGAAC-3′ and 5′-CCGGAGCCATTGTCACACAC-3′; TGF-β1: 5′-TGCGCCTGCAGAGATTCAAG-3′ and 5′-AGGTAACGCCAGGAATTGTTGCTA-3′; IL-10: 5′-CAGACCCACATGCTCCGAGA-3′ and 5′-CAAGGCTTGGCAACCCAAGTA-3′; MMP-9: 5′-TCCAGTAGACAATCCTTGCAATGTG-3′ and 5′-CTCCGTGATTCGAGAACTTCCAATA-3′; TIMP-1: 5′-ACAGGTTTCCGGTTCGCCTAC-3′ and 5′-CTGCAGGCAGTGATGTGCAA-3′; and GAPDH: 5′-GGCACAGTCAAGGCTGAGAATG-3′ and 5′-ATGGTGGTGAAGACGCCAGTA-3′.

### Protein extraction and western blot analysis

After being treated with 0, 0.5, 1.0 or 1.5 mg/ml pirfenidone for 48 h, cells were lysed with lysis buffer [1% Triton X-100, 20 mM HEPES (pH 7.5), 150 mM NaCl, 1 mM EDTA, 1 mM EGTA, 1 mM DTT, 1 mM β-glycerol-phosphate, 1 mM Na_3_VO_4_, 1 mM PMSF, and 10 µg/ml each of leupeptin, aprotinin, and pepstatin]. The cell extract protein concentration was quantified by the BCA assay. Equal protein (30 µg) amounts of the lysates were separated by 4–12% gradient SDS-PAGE and transferred to a nitrocellulose membrane. The membranes were blocked with 5% skimmed milk in Tris-buffered saline with Tween-20, and subsequently incubated overnight at 4°C with anti-α-SMA monoclonal antibody (1∶2000), washed, and then incubated with horseradish-peroxidase-conjugated secondary antibody (1∶5000) for 1 h at room temperature. Immunoreactive bands were visualized using enhanced chemiluminescence reagent, and quantified by densitometry with the Bio-Rad Universal Hood and Quantity One software. Protein levels of α-SMA were standardized by comparison with respective levels of β-tubulin.

### Immunocytochemistry and TUNEL assay

CFs were plated on cover slides in six-well culture plates and treated with 0, 0.5, 1.0 or 1.5 mg/ml pirfenidone for 48 h, fixed with 4% paraformaldehyde, and permeabilized with 0.3% Triton X-100. The cells were blocked with 10% normal goat serum, incubated with primary antibodies to Ki67 (1∶50) and α-SMA (1∶500), respectively. After incubation with the primary antibodies, cultures were rinsed in PBS and incubated in either Alexa Fluor 488 or rhodamine-conjugated secondary antibodies. Nuclei were stained with 4′,6-diamidino-2-phenylindole (DAPI). For TUNEL assay, dead cells were detected using the Fluorescein FragEL™ DNA Fragmentation Detection Kit following the manufacturer's instructions. Fluorescent staining was visualized on a BX-61 microscope (Olympus). The labeling index of Ki67 or TUNEL (the percentage of positively stained nuclei) was calculated in 10 random high power fields (×400).

### Gelatin zymography

Relative levels of MMP-9 in cell culture supernatants were determined by zymography. Cell-free culture supernatants were harvested at 48 h after treatment with pirfenidone at different concentrations (0, 0.5, 1.0 and 1.5 mg/ml), samples were stored at –70°C, and their protein concentrations were determined by BCA assay. Equal protein amounts were loaded onto Novex 10% zymogram gels containing 0.1% gelatin and separated under non-reducing conditions. Gels were washed with renaturing buffer for 30 min and incubated at 37°C in developing buffer. After 20 h, gels were stained with the Colloidal Blue Staining Kit according to the protocol. Gels were scanned, and density analysis of the bands was performed using Quantity One software.

### ELISA

Secretion of TGF-β1, IL-10 and TIMP-1 in the culture supernatants of CFs with or without pirfenidone treatment (0, 0.5, 1.0 and 1.5 mg/ml for 48 h) was determined by ELISA using the commercially available kits, according to the manufacturer's instructions. Absorbance was measured at 450 nm using a microplate reader. Results were compared with a standard curve constructed by titrating standards respectively. The cellular protein contents per culture flask were determined with the BCA assay. All forms of concentrations were standardized to respective cellular protein contents, transformed to pg/µg (cellular protein), then expressed as a percentage of the controls.

### Cytotoxicity assay

Cell viability was assessed both by the trypan blue exclusion test and by measuring the release of lactate dehydrogenase (LDH). For trypan blue exclusion test, following treatment with different concentrations of pirfenidone (0, 0.5, 1.0 and 1.5 mg/ml) for the specified time periods (24, 48 and 72 h), CFs were harvested and labeled with trypan blue (0.4% in PBS). Trypan-blue-positive and -negative cells were calculated with a hematocytometer. Trypan-blue-negative cells were regarded as viable cells. The percentage cell viability was calculated using the following formula: % cell viability (viable cell count/total cell count) ×100. In assays investigating LDH activity, after treatment with different concerntrations of pirfenidone, the culture medium was collected and assayed using the LDH Cytotoxicity Detection Kit according to the manufacturer's instructions. Cell death was determined as LDH release, expressed as a percentage of the mean absorbance measured in untreated control cultures.

### Statistical analysis

Data are expressed as the mean ± SEM of at least three independent experiments, unless otherwise stated. Differences among groups were tested by one-way ANOVA. A value of P<0.05 was considered statistically significant.

## Results

### Effects of pirfenidone on CF proliferation

**Figure 1:**
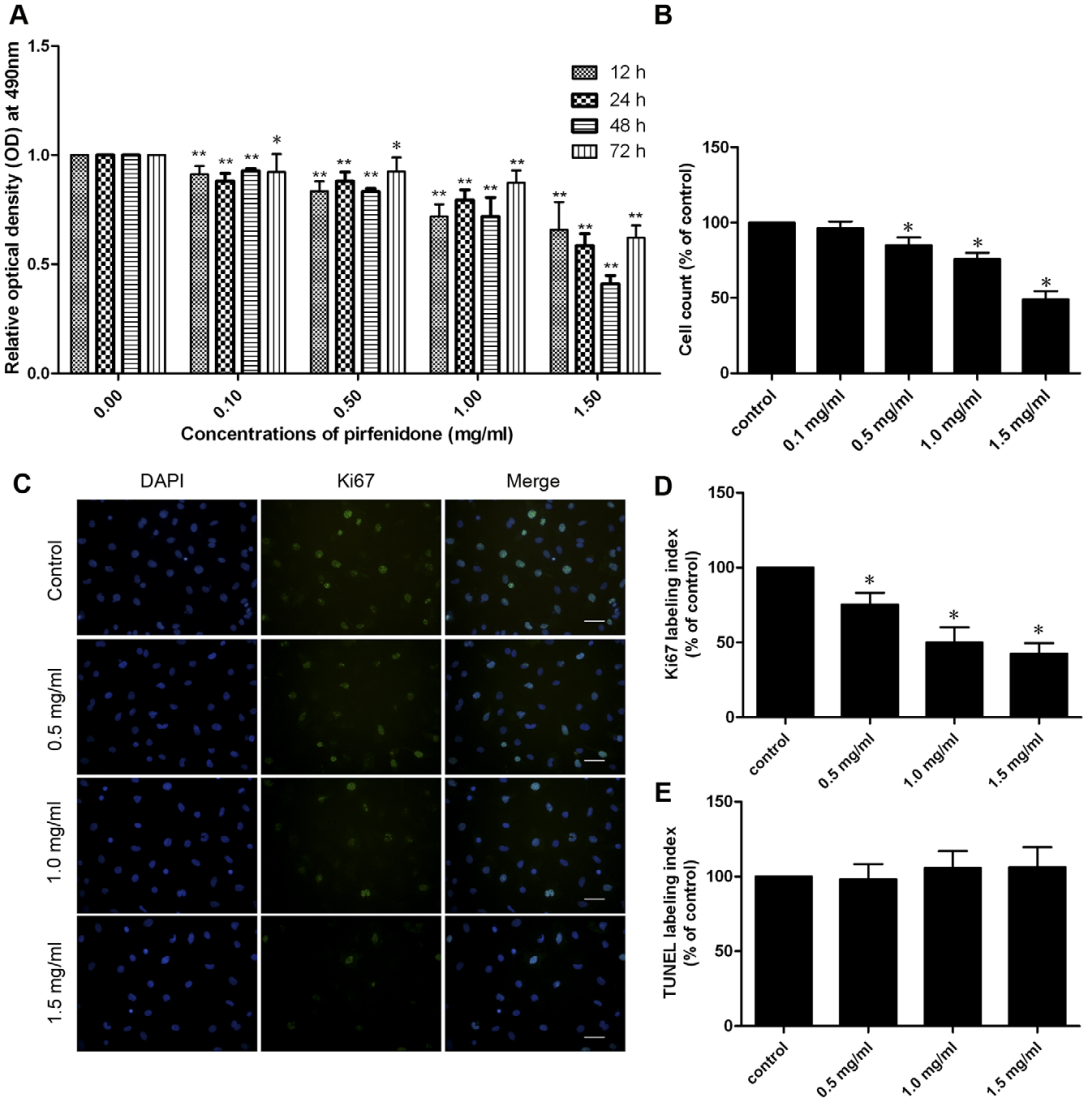
Pirfenidone inhibited proliferation of CFs. A. MTS assay. Cells with 10% FCS were treated with 0, 0.1, 0.5, 1.0 or 1.5 mg/ml pirfenidone for 12, 24, 48 or 72 h. Data are expressed as mean ± SEM (n = 5). B. Cells with 10% FCS were treated with 0, 0.1, 0.5, 1.0 or 1.5 mg/ml pirfenidone for 48 h. Cells were resuspended and counted under the microscope. Data represent mean ± SEM. C. Representative examples of Ki67 staining in CFs, scale bar = 50.0 µm. D and E. Ki67 or TUNEL labelling index of the different groups expressed as a percentage of the control. Data represent mean ± SEM. Representative examples of TUNEL staining in CFs are shown in Figure S1. *P<0.05, **P<0.01, versus control.

MTS assay (Figure 1A) showed that, at concentrations of 0.1, 0.5, 1.0 and 1.5 mg/ml, pirfenidone inhibited proliferation of CFs in a dose- and time-dependent manner compared with the control group. A time course experiment with 1.5 mg/ml pirfenidone suggested that the maximal inhibitory response was observed after 48 h treatment. The significantly inhibited cell proliferation of CFs after treatment with different concentrations of pirfenidone for 48 h was also validated by the cell counting results (Figure 1B). Further assessment of proliferative activity of CFs was performed by immunostaining of Ki67, a cellular marker for proliferation (Figure 1C). Quantification showed a significant decrease in proliferating CFs following pirfenidone treatment (Figure 1D), with no significant increase in apoptosis compared with control (Figure 1E and Figure S1).

### Effects of pirfenidone on α-SMA expression and collagen contraction

**Figure 2:**
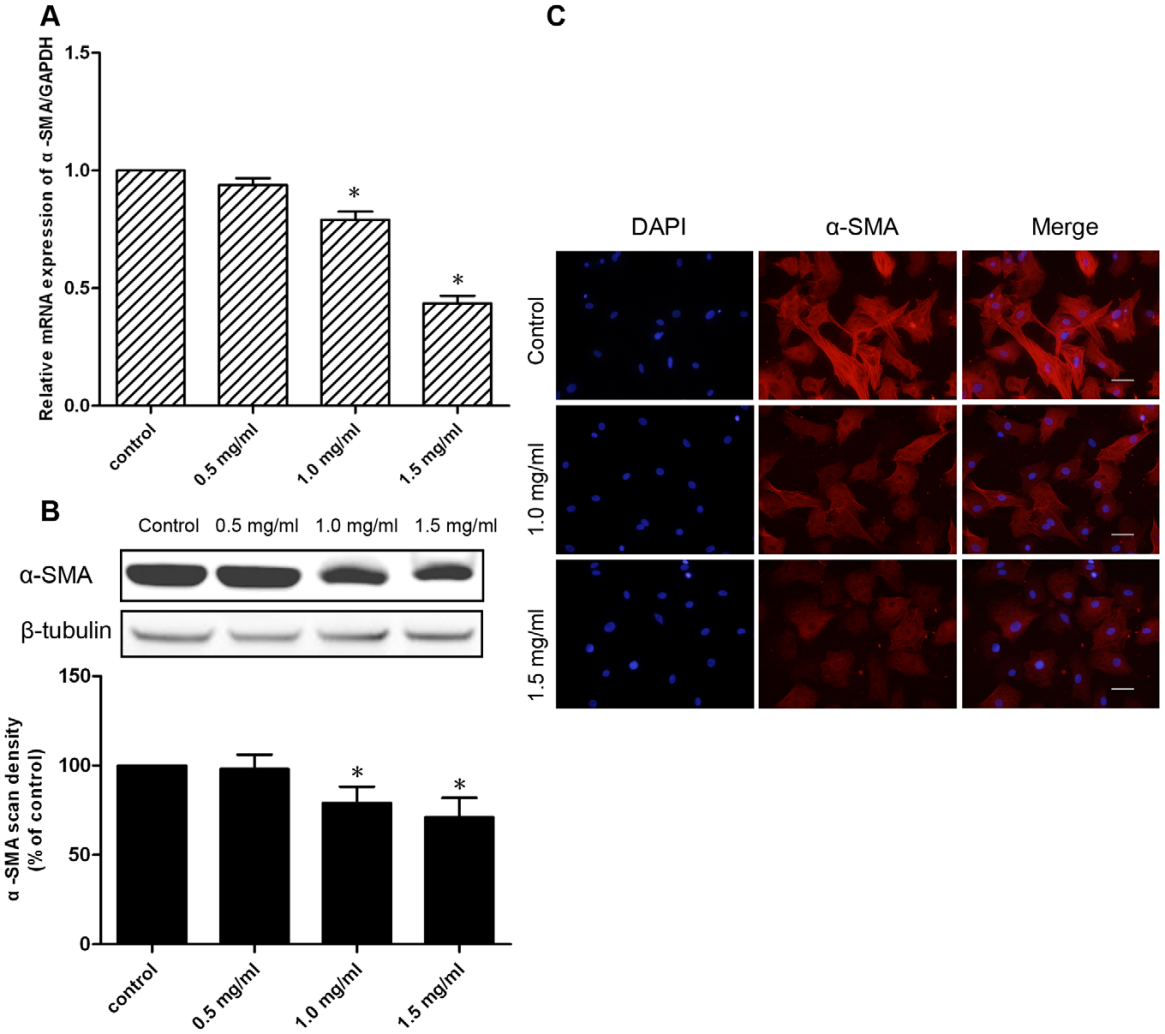
Effects of pirfenidone on α-SMA expression. CFs were treated with 0.5, 1.0 or 1.5 mg/ml pirfenidone for 48 h. A. Effects of pirfenidone on α-SMA mRNA expression of CFs were determined by real-time PCR. B. Effects of pirfenidone on α-SMA protein expression in CFs were determined by western blotting. Upper panel shows a representative immunoblot, and the lower panel the pooled relative values of densitometric scanning. C. Effects of 1.0 or 1.5 mg/ml pirfenidone on α-SMA expression and morphological changes of CFs were determined by immunofluorescence staining with anti-α-SMA antibody. Scale bar = 50.0 µm. Data are the mean ± SEM, *P<0.05 versus control.

Myofibroblast differentiation is perceived to be important for the development of cardiac fibrosis. Therefore, we investigated the effects of pirfenidone on CF differentiation. The expression and organization of α-SMA are hallmarks of myofibroblast differentiation. Real-time PCR and western blotting were used to detect α-SMA expression after 48 h treatment with 0.5, 1.0 or 1.5 mg/ml pirfenidone, which showed that doses of 1.0 and 1.5 mg/ml significantly inhibited α-SMA expression at both the mRNA and protein levels in CFs (Figure 2A, B).

In addition, effects of pirfenidone on myofibroblast differentiation were further investigated by immunofluorescence. Staining for α-SMA was used to visualize actin stress filaments and cellular morphological changes in CFs. Immunofluorescent analysis revealed that 1.0 and 1.5 mg/ml pirfenidone markedly decreased formation of stress fibers and brightness of α-SMA staining in CFs induced by 10% serum (Figure 2C).

**Figure 3:**
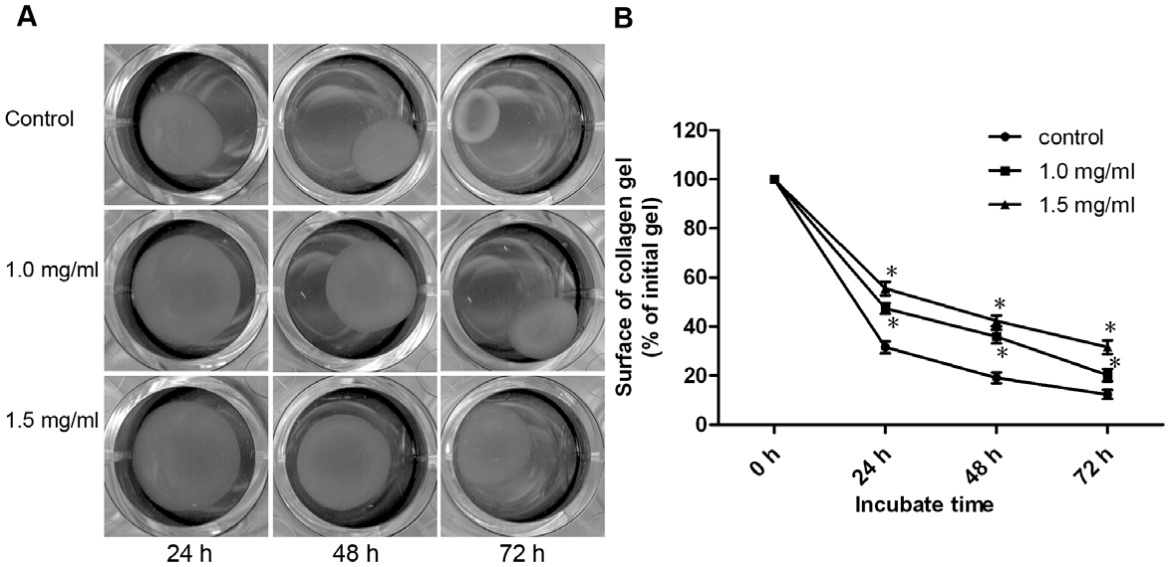
Pirfenidone inhibited the ability of CFs to contract collagen gels. CFs were seeded in collagen lattice in the absence or presence of pirfenidone at concentrations of 1.0 and 1.5 mg/ml. Cell contractility was assessed by measuring the reduction in the surface area of the collagen gel discs for the times shown (1–3 days). A. Photographs of one representative experiment. B. Graphic representation of the mean ± SEM. *P<0.05 versus control.

To determine the effect of pirfenidone on ECM contraction induced by CFs, CFs were seeded in free-floating collagen gels and incubated in the presence or absence of pirfenidone for 24, 48 and 72 h. Pirfenidone at 1.0 and 1.5 mg/ml significantly inhibited collagen lattice contraction by CFs, when compared with the control group (Figure 3).

### Effects of pirfenidone on CF migration

**Figure 4:**
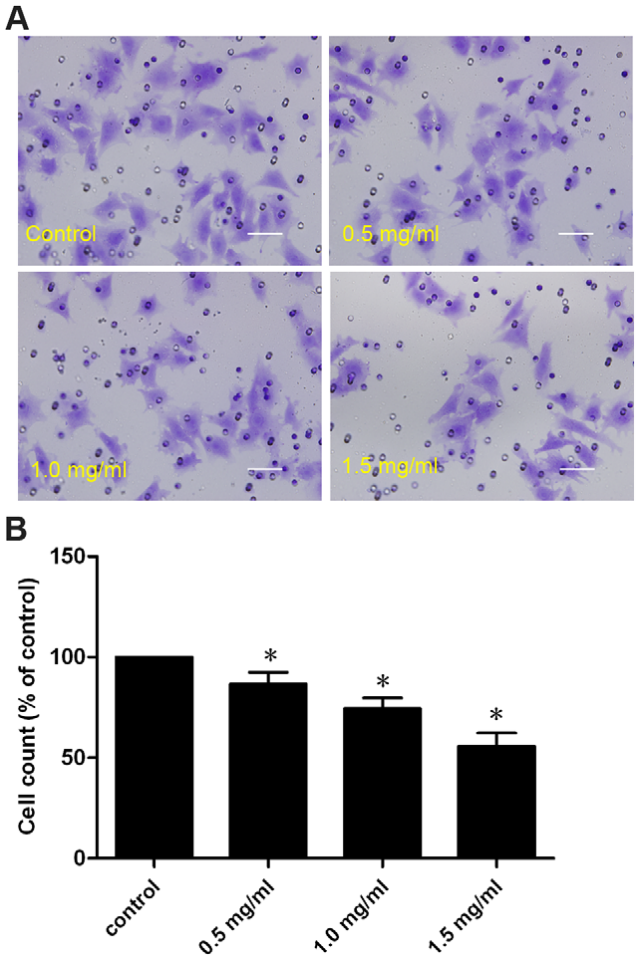
Pirfenidone inhibited migration of CFs. A. Representative images of CFs migrating to underside of membrane after 24 h in presence or absence of 0.5, 1.0 or 1.5 mg/ml pirfenidone. Pores in the membrane are visible as dark circles. Scale bar = 50.0 µm. B. Pooled data expressed as percentage migration observed with controls. Data represent mean ± SEM, *P<0.05 versus control.

To examine the effects of pirfenidone on CF migration, a modified Boyden chamber assay was used, in which cell culture inserts were coated with a thin layer of the Matrigel basement membrane matrix, to mimic the *in vivo* situation of cellular migration, which includes two distinct phases: degradation of ECM, followed by cellular migration towards a chemotactic stimulus. After 24 h incubation, fewer cells were observed on the bottom side of the polyethylene terephthalate membranes in pirfenidone-treated groups (0.5, 1.0 and 1.5 mg/ml) when compared with the control group. This indicated that pirfenidone significantly impeded the capability of CFs to invade across the layer of the Matrigel matrix (Figure 4).

### Effects of pirfenidone on MMP-9, TIMP-1 synthesis and secretion

**Figure 5:**
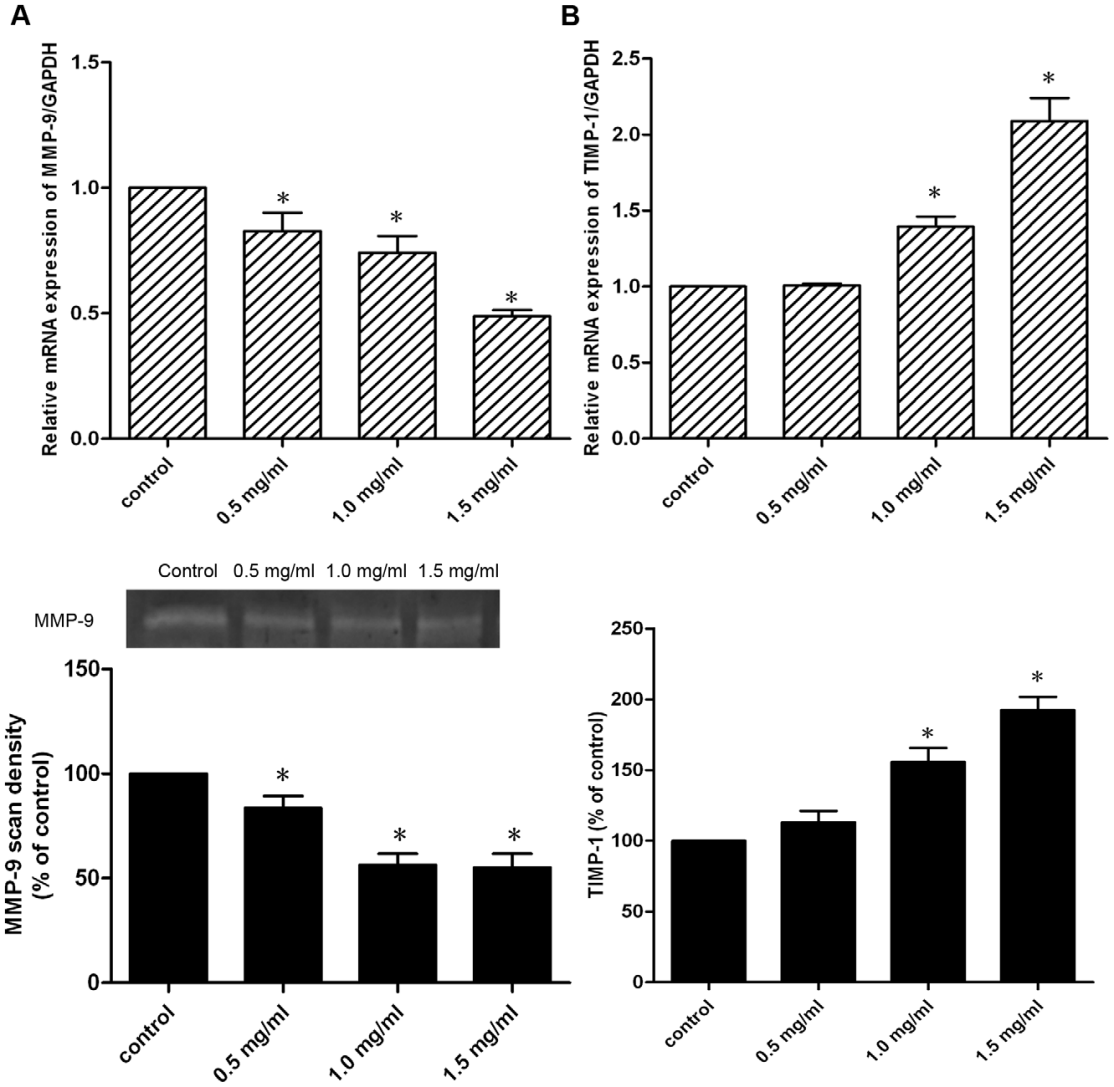
Effects of pirfenidone on MMP-9 and TIMP-1 synthesis and secretion. CFs were treated with 0.5, 1.0 or 1.5 mg/ml pirfenidone for 48 h. A. Upper panel: MMP-9 mRNA expression determined by real-time PCR. Lower panel: MMP-9 activity determined by gelatin zymography. Representative gelatin zymogram is shown. Graphic representation of pooled data depicts densitometric analysis of MMP-9 band intensity. B. Upper panel: TIMP-1 mRNA expression determined by real-time PCR. Lower panel: TIMP-1 protein secretion determined by ELISA. Data represent mean ± SEM, *P<0.05 versus control.

The migratory activity of CFs is believed to be directly proportional to the MMP activity and inversely proportional to the TIMP activity, hence the synthesis and secretion of MMP-9 and TIMP-1 were further investigated in CFs treated with or without pirfenidone. Real-time PCR showed that treatment with pirfenidone resulted in a dose-dependent decrease in MMP-9 mRNA expression, whereas TIMP-1 mRNA levels were increased by pirfenidone dose-dependently. In addition, gelatin zymography indicated that the inhibitory effect of pirfenidone on MMP-9 activity in cell culture supernatants was dose-dependent, with maximal MMP-9 inhibition at 1.5 mg/ml for 48 h incubation. ELISA also showed that pirfenidone stimulated TIMP-1 secretion in cell culture supernatants in a dose-dependent manner (Figure 5).

### Effects of pirfenidone on TGF-β1 and IL-10 synthesis and secretion

**Figure 6:**
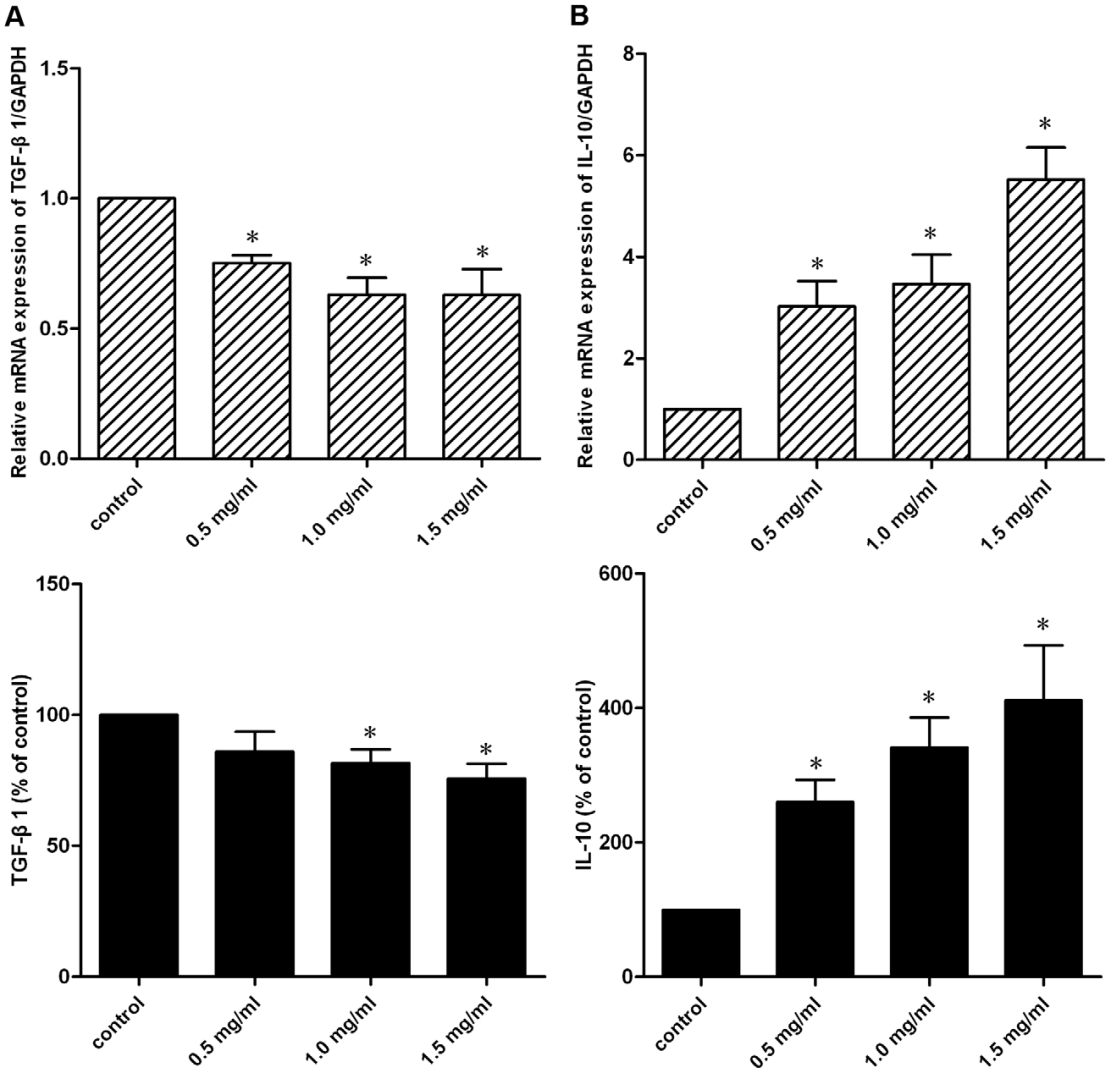
Effects of pirfenidone on TGF-β1 and IL-10 synthesis and secretion. CFs were treated with 0.5, 1.0 or 1.5 mg/ml pirfenidone for 48 h. A. Upper panel: TGF-β1 mRNA expression determined by real-time PCR. Lower panel: TGF-β1 protein secretion determined by ELISA. B. Upper panel: IL-10 mRNA expression determined by real-time PCR. Lower panel: IL-10 protein secretion determined by ELISA. Data represent mean ± SEM, *P<0.05 versus control.

Expression and secretion of TGF-β1 and IL-10 by CFs was investigated at both mRNA and protein levels. As shown in the upper panel of Figure 6, exposure of CFs to pirfenidone led to a decrease in TGF-β1 transcription, and on the contrary, IL-10 mRNA expression levels were elevated. Next, the secretion of TGF-β1 and IL-10 from the cell culture supernatants was examined by ELISA. As shown in the lower panel of Figure 6, adding pirfenidone to CFs resulted in decreased TGF-β1 secretion but enhanced IL-10 secretion significantly. Additionaly, as it is well-recognized that Ang II is an effective inducer of TGF-β1, we further investigated the effects of pirfenidone in Ang II-stimulated CFs, and results showed that pirfenidone also attenuated Ang II-stimulated TGF-β1 expression significantly (Figure S2).

### Toxicology of pirfenidone

**Figure 7:**
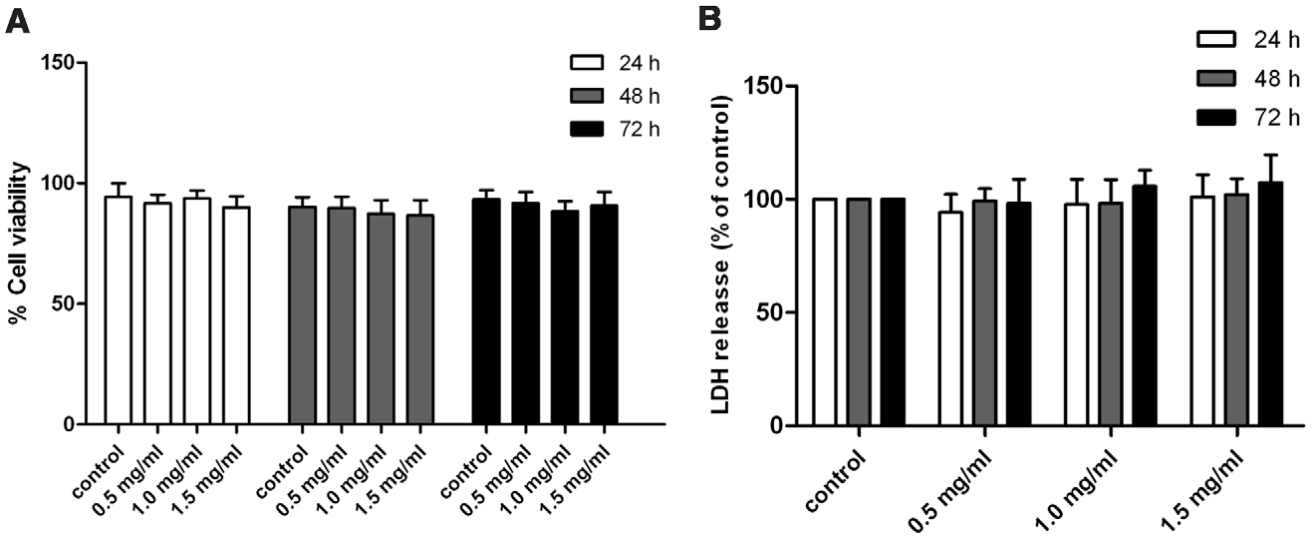
Cytotoxicity effects of pirfenidone on CFs. Following treatment with different concentrations of pirfenidone (0, 0.5, 1.0 and 1.5 mg/ml) for the specified time periods (24, 48 and 72 h). Trypan blue exclusion test (A) or LDH assay (B) was carried out to examine the cytotoxicity effects of pirfenidone on CFs. Data represent mean ± SEM. P>0.05 versus control.

To exclude the possibility that the antifibrotic effects of pirfenidone were mediated by cellular toxicity, the trypan blue exclusion test and LDH release assay were carried out to examine the viability of CFs after administration of pirfenidone at different concentrations and time periods. Results indicated that at the tested concentrations and time periods, pirfenidone had no significant cytotoxicity effect on cultured CFs (Figure 7). This suggests pirfenidone might exert its antifibrotic effects in a non-cytotoxic manner.

## Discussion

The present study was designed to determine whether pirfenidone has direct cellular effects on CF functions that are important in the cardiac remodeling process. We demonstrated that pirfenidone: (1) inhibited proliferation of CFs; (2) inhibited myofibroblast differentiation, collagen contractility and migratory ability of CFs; (3) reduced the synthesis and secretion of MMP-9 and increased that of TIMP-1, i.e., decreased the ratio of MMP9/TIMP-1 in CFs; and (4) decreased the synthesis and secretion of TGF-β1 but enhanced that of IL-10 in CFs.

Pirfenidone is a novel, broad spectrum antifibrotic agent. Its antifibrotic effect was first described in 1995 in a hamster model of bleomycin-induced lung fibrosis [29], and since then, its beneficial effects have been confirmed in several animal models with fibrosing diseases in different organs. It has been reported that pirfenidone inhibits cardiac fibrosis in several animal models, and in one recent study in a rat model of myocardial infarction [15], it was shown that pirfenidone is able to improve cardiac function, reduce infarct dense scarring, and attenuate left ventricular fibrosis. Together with results from earlier publications [13], [16], [17], there is strong evidence that pirfenidone has antifibrotic effects during adverse cardiac remodeling. However, although results from these *in vivo* studies suggest that CFs represent the major targets of pirfenidone, the effects of pirfenidone on isolated and cultured CFs are at present largely unknown.

CF proliferation is vital for ventricular remodeling. The inhibitory effect of pirfenidone on proliferation has been illustrated in a variety of cell types *in vitro* [18]–[23]. In the present study, by using different methods, we showed that pirfenidone inhibited CF proliferation in a dose- and time-dependent manner. Furthermore, at the tested doses, pirfenidone did not induce any significant changes in the viability of cells treated with pirfenidone compared with that in control cultures, as detected by TUNEL assay, trypan blue exclusion test and LDH assay. Therefore, the antiproliferation effect was probably not due to a direct cytotoxic effect of pirfenidone. CFs represent the largest class of cells residing in the heart, and the proliferation of CFs is the main characteristic of myocardial fibrosis. Hence, these results indicate the potential effectiveness of pirfenidone in the treatment of cardiac fibrosis.

The phenotypic transformation of CFs to myofibroblasts is perceived to be another key event in the wound-healing and remodeling processes. Myofibroblasts are highly active cells that express α-SMA, and exhibit increased proliferative, migratory and secretory properties. Under normal circumstances, the myofibroblasts are scavenged from the repaired wound site by apoptosis [30], [31]. However, persistence of myofibroblasts can facilitate hypertrophic scarring and fibrosis, which results in myocardial stiffness and impairment of cardiac function [32], [33]. Prevention of myofibroblast differentiation might therefore represent a potential target for therapies aimed at limiting fibrosis in the heart. In this study, we found that pirfenidone attenuated the α-SMA expression in CFs, and decreased their collagen contractility.

Another important step in the remodeling process is activation of MMPs that are necessary for degrading the basement membrane matrix, a prerequisite for both cell proliferation and migration *in vivo*. TIMPs are locally synthesized proteins that bind to active MMPs and thereby regulate net proteolytic activity, therefore the MMP–TIMP axis plays a crucial role in cardiac remodeling. Cardiac MMP-9 activity is increased in animal models of heart injury [34], [35] and in HF patients [36], [37], and targeted deletion of MMP-9 attenuates myocardial remodeling in mice [38]. In a recent study [39], by constructing a recombinant protein encoding catalytic domain of MMP-9, it was shown that MMP-9 induces CFs migration, differentiation and cytokine secretion directly. Previously, it has been reported [16] that pirfenidone attenuates MMP-9 expression in the atrial tissue in a canine chronic HF model. Our results showed that addition of pirfenidone to CFs significantly decreased MMP-9 mRNA expression and activity. In addition, elevated mRNA and protein levels of TIMP-1 were observed after pirfenidone treatment in this study. It has been demonstrated that MMP levels are high and TIMP levels are low in HF patients [37], [40], [41], and in particular, the MMP-9/TIMP-1 ratio is increased in HF patients [36]. This disparity between MMP and TIMP levels favors a persistent MMP activation state within the myocardium and probably contributes to cardiac remodeling in the setting of developing chronic HF. The opposite regulatory effects of pirfenidone on MMP-9 and TIMP-1 in CFs imply that pirfenidone is able to normalize the balance between MMPs and TIMPs, which may serve as an important mechanism underlying its cardioprotective effects.

TGF-β1 is now the most well-investigated profibrotic cytokine, it's crucial role in cardiac remodeling has been well recognized [42] and *in vitro* studies have confirmed that it can enhance myofibroblast differentiation of CFs significantly [43], [44]. Many studies have shown that pirfenidone can reduce production of TGF-β1 *in vitro* [20], [45] and *in vivo* [46]–[49], and one study in particular has demonstrated that pirfenidone prevents congestive-HF-induced TGF-β1 overexpression in the atrium [16]. In the present study, we found similar results: pirfenidone treatment reduced both the synthesis and secretion of TGF-β1 in cultured CFs. In a previous study using a murine model of endotoxic shock [50], it has been shown that the production of IL-10, which is recognized as an anti-inflammatory cytokine, was markedly enhanced after administration of pirfenidone. In this study, we found that the synthesis and secretion of IL-10 were also increased in cultured CFs, as a result of pirfenidone treatment. The antifibrotic effects of IL-10 have been reported in different animal models of liver [51], airway [52] and kidney [53] fibrosis, in addition, it has been shown that IL-10 inhibits proliferation and α-SMA expression in cultured neonatal CFs [54]. Thus, augmentation of IL-10 expression might be another mechanism that underlies the antifibrotic effects of pirfenidone. Taken together, we illustrated that pirfenidone could further exert its antifibrotic effects by modulating cytokine secretion in CFs, suppressing cytokine TGF-β1 production, but on the other hand, enhancing that of IL-10.

In summary, the results of the present study suggest that pirfenidone is able to exert its antifibrotic effect in CFs in a direct manner, and acting at both a cellular and a molecular level. At a cellular level, pirfenidone inhibited CF proliferation, contraction of collagen, and migration; and at a molecular level, pirfenidone reduced α-SMA expression, decreased the MMP-9/TIMP-1 ratio, and suppressed profibrotic cytokine TGF-β1 production, but enhanced that of anti-inflammatory cytokine IL-10. Although the detailed mechanisms underlying these effects remain to be determined, it is unlikely that they are the results of cytotoxicity. Coupled with the results of previous *in vivo* studies, we propose that pirfenidone may be a promising candidate for the treatment of cardiac fibrosis during pathological myocardial remodeling.

## Supporting Information

Figure S1 **Representative examples of TUNEL staining in CFs.** Cells with 10% FCS were treated with 0, 0.5, 1.0 or 1.5 mg/ml pirfenidone for 48 h. Nuclei were stained with DAPI (blue), no significant increase in TUNEL staining (green) was observed in pirfenidone-treated groups. Scale bar = 50.0 µm.
(TIF)

Figure S2 **Effect of pirfenidone on Ang II-induced TGF-β1 expression.** CFs were cotreated with 100nM Ang II and different concentrations of pirfenidone (0, 0.5, 1.0 or 1.5mg/ml) for 24 h. A. TGF-β1 mRNA expression determined by real-time PCR. B. TGF-β1 protein secretion determined by ELISA. Data are the mean ± SEM, *P<0.05 versus control; #P<0.05 versus Ang II-stimulated cells.
(TIF)
