# Supplementary figures and images for: In Vitro Effects of Pirfenidone on Cardiac Fibroblasts: Proliferation, Myofibroblast Differentiation, Migration and Cytokine Secretion
Source: PLoS One. 2011 Nov 23;6(11):e28134. doi: 10.1371/journal.pone.0028134 (PMC3223242; doi:10.1371/journal.pone.0028134)

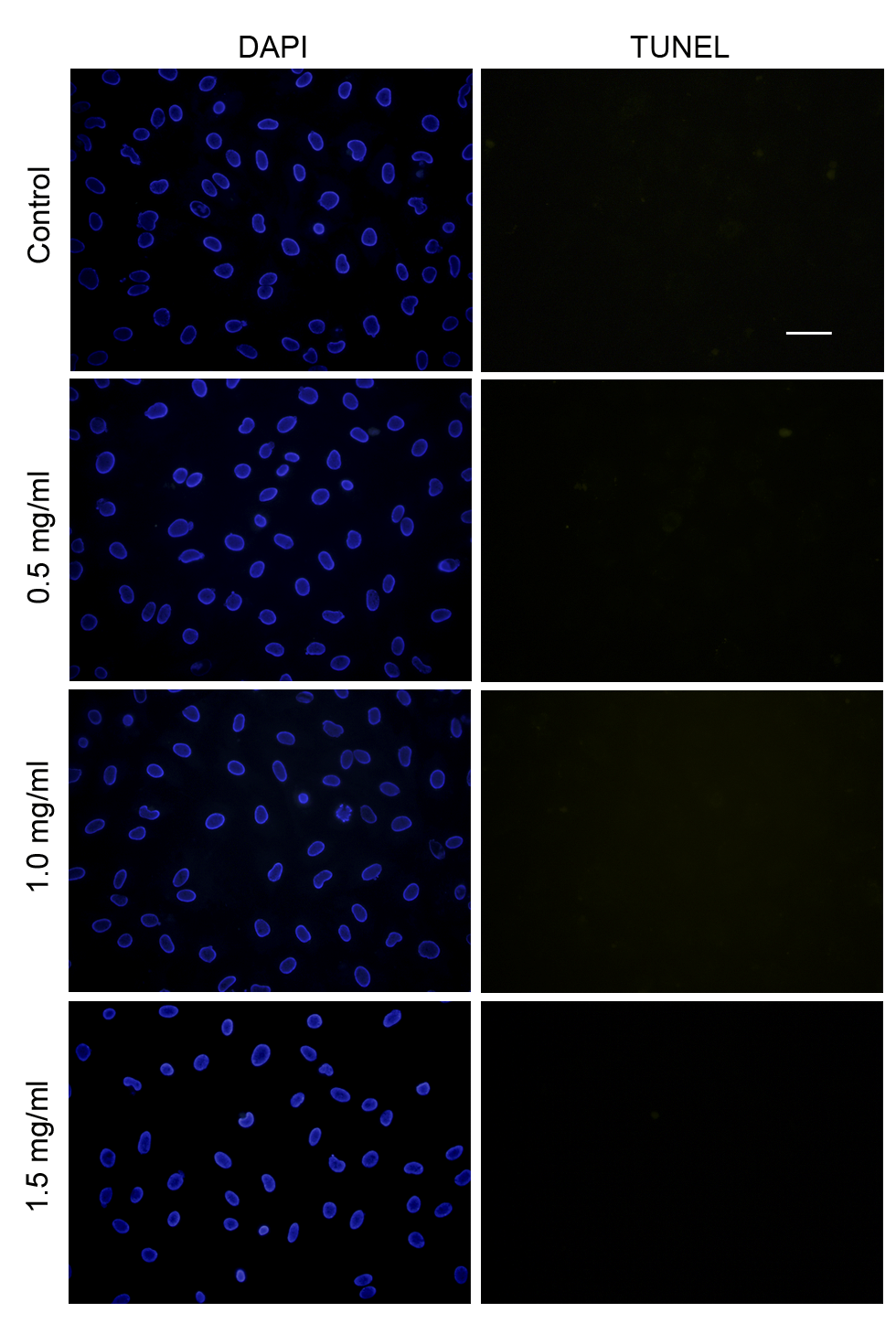

Supplement: Figure S1 — Representative examples of TUNEL staining in CFs. Cells with 10% FCS were treated with 0, 0.5, 1.0 or 1.5 mg/ml pirfenidone for 48 h. Nuclei were stained with DAPI (blue), no significant increase in TUNEL staining (green) was observed in pirfenidone-treated groups. Scale bar = 50.0 µm. (TIF) [file pone.0028134.s001.tif]

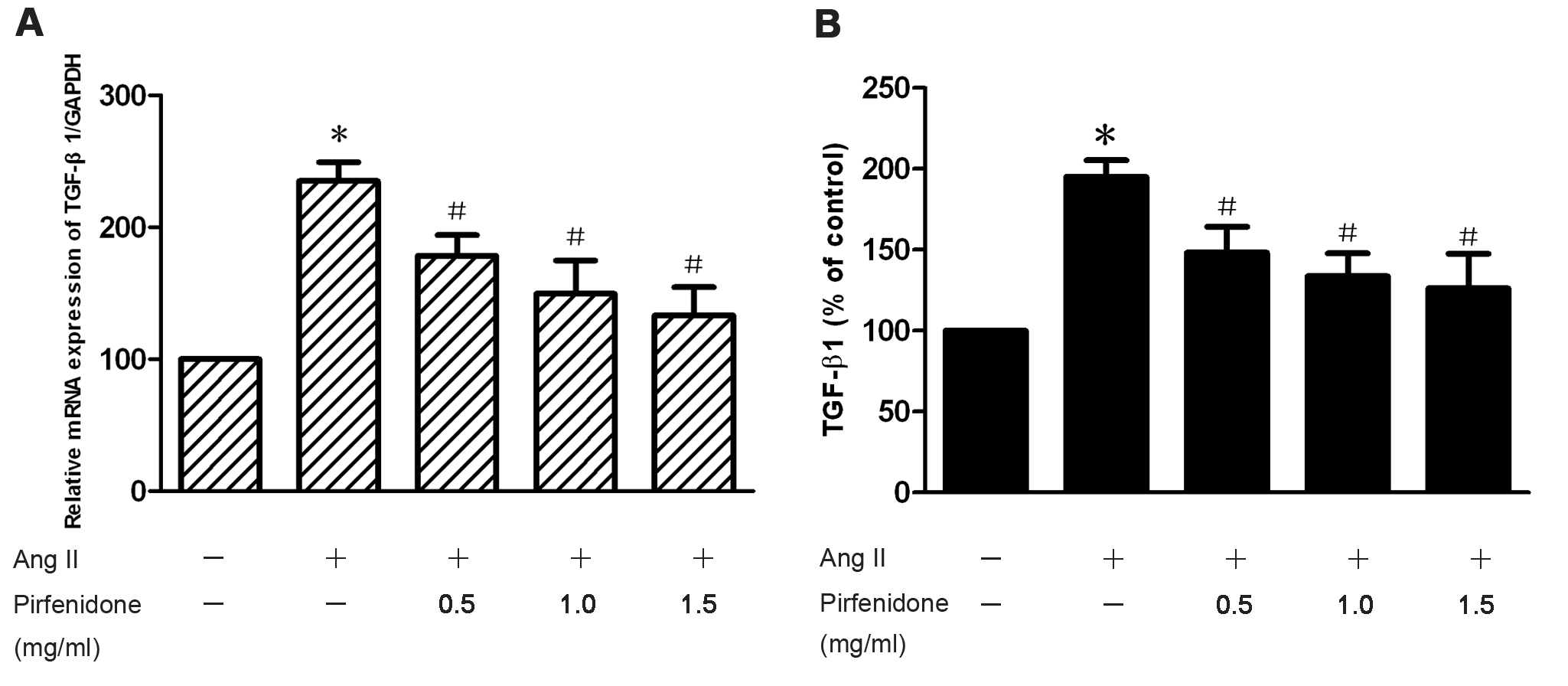

Supplement: Figure S2 — Effect of pirfenidone on Ang II-induced TGF-β1 expression. CFs were cotreated with 100nM Ang II and different concentrations of pirfenidone (0, 0.5, 1.0 or 1.5mg/ml) for 24 h. A. TGF-β1 mRNA expression determined by real-time PCR. B. TGF-β1 protein secretion determined by ELISA. Data are the mean ± SEM, *P<0.05 versus control; #P<0.05 versus Ang II-stimulated cells. (TIF) [file pone.0028134.s002.tif]
